# No Survival Benefit from Adding Cetuximab or Panitumumab to Oxaliplatin-Based Chemotherapy in the First-Line Treatment of Metastatic Colorectal Cancer in KRAS Wild Type Patients: A Meta-Analysis

**DOI:** 10.1371/journal.pone.0050925

**Published:** 2012-11-30

**Authors:** Si-wei Zhou, Yuan-yuan Huang, Ying Wei, Zhi-min Jiang, Yuan-dong Zhang, Qiong Yang, De-rong Xie

**Affiliations:** 1 Department of Oncology, Sun Yat-sen Memorial Hospital, Key Laboratory of Malignant Tumor Gene Regulation and Target Therapy of Guangdong Higher Education Institutes, Sun Yat-sen University, Guangzhou, Guangdong, People’s Republic of China; 2 State Key Laboratory of Oncology in South China, VIP Region, Sun Yat-sen University Cancer Center, Guangzhou, Guangdong, People’s Republic of China; 3 Cancer Center, Guangdong General Hospital, Guangdong Academy of Medical Sciences, Guangzhou, Guangdong, People’s Republic of China; Baylor University Medical Center, United States of America

## Abstract

**Background:**

The efficacy of combined therapies of oxaliplatin-based chemotherapy and anti-epidermal growth factor receptor (anti-EGFR) monoclonal antibodies (MAbs) remains controversial in colorectal cancer (CRC). The aim of this study is to estimate the efficacy and safety of adding cetuximab or panitumumab to oxaliplatin-based chemotherapy in the first line treatment in KRAS wild type patients with metastatic colorectal cancer (mCRC) through meta-analysis.

**Methods:**

Medline, EMBASE, and Cochrane library, American Society of Clinical Oncology (ASCO) and European Society for Medical Oncology (ESMO) were searched. Eligible studies were randomized controlled trials (RCTs) which evaluated oxaliplatin-based chemotherapy with or without anti-EGFR drugs (cetuximab or panitumumab) in untreated KRAS wild type patients with mCRC. The outcomes included overall survival (OS), progression-free survival (PFS), overall response rate (ORR) and toxicities. Hazard ratios (HR) and risk ratio (RR) were used for the meta-analysis and were expressed with 95% confidence intervals.

**Results:**

This meta-analysis included four RCTs with 1270 patients, and all of the patients were administered oxaliplatin-based chemotherapy regimens with or without anti-EGFR MAbs. The result of heterogeneity of OS was not significant. Compared with chemotherapy alone, the addition of cetuximab or panitumumab didn’t result in significant improvement in OS (HR = 1.00, 95%CI [0.88, 1.13], P = 0.95) or PFS (HR = 0.86, 95%CI [0.71, 1.04], P = 0.13). The subgroup analysis of cetuximab also revealed no significant benefit in OS (HR = 1.02, 95%CI [0.89, 1.18], P = 0.75) or in PFS (HR = 0.87, 95%CI [0.65, 1.17], P = 0.36). Patients who received combined therapy didn’t have a higher ORR (Risk Ratio = 1.08, 95%CI [0.86, 1.36]). Toxicities slightly increased in anti-EGFR drugs group.

**Conclusions:**

The addition of cetuximab or panitumumab to oxaliplatin-based chemotherapy in first-line treatment of mCRC in wild type KRAS population did not improve efficacy in survival benefit and response rate. More RCTs are warranted to evaluate the combination of chemotherapy and targeted therapy.

## Introduction

Colorectal cancer is the third most frequently diagnosed cancer in males and the second in females. In contrast with high incidence, the death rate of CRC was decreasing in several western countries owing to improved treatment, increased awareness and early detection [1]. As more and more active drugs have been introduced into the treatment of mCRC, including chemotherapy drugs and targeted therapy drugs, the median OS of patients with mCRC has been improved considerably [2], [3]. Serving as the foundation of chemotherapy backbone in advanced CRC, irinotecan and oxaliplatin show confirmed activity in terms of survival benefit. Anti-epidermal growth factor receptor (anti-EGFR) monoclonal antibodies (MAbs) also have activity in the treatment of mCRC both as monotherapy and in combination with irinotecan-based therapy, proven by RCTs [4], [5].

However, the results of clinical trials about addition of anti-EGFR MAb to oxaliplatin-based chemotherapy seem to get fewer consensuses than irinotecan-based chemotherapy. The interaction between oxaliplatin and cetuximab or panitumumab remains unknown. Therefore, we performed a meta-analysis in order to evaluate the survival benefit in these combined therapies.

**Figure 1:**
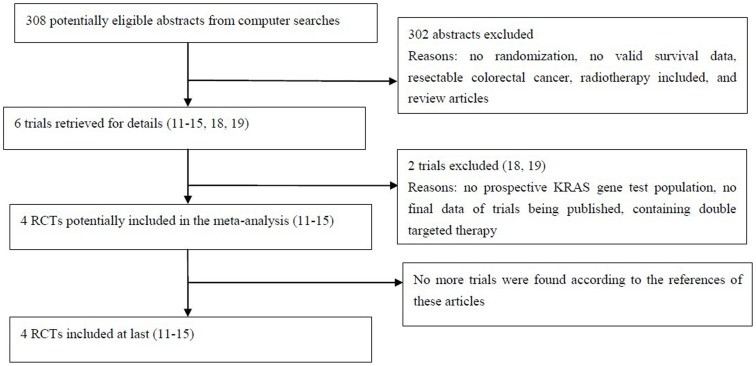
The flow chart.

The KRAS gene status is confirmed as a predictive marker of anti-EGFR MAb therapy in mCRC. Patients with KRAS gene mutation do not benefit from cetuximab or panitumumab, demonstrated in a number of retrospective and prospective studies [6], [7]. The National Comprehensive Cancer Network (NCCN, website http://www.nccn.org/index.asp) Clinical Practice Guideline in Oncology Colon Cancer 2011 version 1 and the American Society of Clinical Oncology (ASCO) 2009 review concluded that only patients with KRAS wild type gene can receive therapy with anti-EGFR agents [8]. In our study, the result and analysis of KRAS mutant CRC were excluded regarding the reasons mentioned above. The aim of this study is to analyze and discuss the efficacy and toxicities of the addition of cetuximab or panitumumab to oxaliplatin-based chemotherapy in the first-line treatment of mCRC, restricting to KRAS wild type patients.

## Methods

### Selection Criteria

#### Types of study

This analysis included all phase III or II randomized controlled trials.

**Table 1:** Randomized controlled trials (OXA+anti-EGFR vs OXA alone).

Studies	Intervention	Patients (KRASwild typepopulation)	Median follow-up(mo)	Median OS and95%CI(mo)	HR and 95%CI for OS	Median PFSand 95%CI(mo)	HR and 95%CI for PFS	ORR	KRAS test	Jadadscore
COIN [13]	FOLFOX/XELOX	367	21	17.9(no report)	1.04 [0.88, 1.22]	8.6(no report)	0.96 [0.82, 1.12]	57%	Prospective	3
	FOLFOX/XELOX+Cetuximab	362	23	17.0(no report)		8.6(no report)		64%		
Nordic VII [14]	FLOX	97	No report	22.0(17.9 to 26.1)	1.14 [0.80, 1.62]	8.7(7.4 to 9.9)	1.07 [0.79, 1.45]	47%	Prospective	3
	FLOX+Cetuximab	97	No report	20.1(14.5 to 25.7)		7.9(6.3 to 9.5)		46%		
OPUS [11], [12]	FOLFOX4	97	No report	18.5(no report)	0.85 [0.60, 1.22]	7.2(no report)	0.57 [0.38, 0.86]	37%	Retrospective	3
	FOLFOX4+Cetuximab	82	No report	22.8(no report)		8.3(no report)		59%		
PRIME [15], [16]	FOLFOX4	325	12.5	19.7(17.6 to 22.7)	0.88 [0.73, 1.06]	8.6(7.5 to 9.5)	0.80 [0.67, 0.95]	48%	Prospective	3
	FOLFOX4+Panitumumab	331	13.2	23.9(20.3 to 27.7)		10.0(9.3 to 11.4)		55%		

FOLFOX in COIN study: oxaliplatin 85 mg/m^2^ on day 1, L-folinic acid 175 mg or D,L-folinic acid 350 mg on day 1, FU 400 mg/m^2^ bolus and FU 2400 mg/m^2^ infusion over 46 h. Every 2 weeks.

XELOX in COIN study: oxaliplatin 130 mg/m^2^ on day 1, capecitabine 850 mg/m^2^ twice a day on day 1 to 14. Every 3 weeks.

Cetuximab in COIN study: an initial dose of 400 mg/m^2^ and thereafter 250 mg/m^2^. Every week.

FLOX in NORDIC VII study: oxaliplatin 85 mg/m^2^ on day 1, FU 500 mg/m^2^ bolus +FA 60 mg/m^2^ bolus on days 1 and 2. Every 2 weeks.

Cetuximab in NORDIC VII study: an initial dose of 400 mg/m^2^ and thereafter 250 mg/m^2^. Every week.

FOLFOX4 in OPUS study: oxaliplatin 85 mg/m^2^ on day 1, leucovorin 200 mg/m^2^ followed by FU 400 mg/m^2^ bolus and 600 mg/m^2^ 22-hour continuous infusion on days 1 and 2. Every 2 weeks.

Cetuximab in OPUS study: an initial dose of 400 mg/m^2^ and thereafter 250 mg/m^2^. Every week.

FOLFOX4 in PRIME study: oxaliplatin 85 mg/m^2^ on day 1, leucovorin 200 mg/m^2^ followed by FU 400 mg/m^2^ bolus and 600 mg/m^2^ 22-hour continuous infusion on days 1 and 2. Every 2 weeks.

Panitumumab in PRIME study: 6 mg/kg. Every 2 weeks.

#### Types of participants

The meta-analysis included patients with mCRC. Eligible patients for the study were ≥18 years old; had histologically or cytologically confirmed mCRC which were previously untreated or no chemotherapy within 6 months before randomization. KRAS wild type gene was also required. Other criteria included Eastern Cooperative Oncology Group (ECOG) performance status of 0 to 2; adequate bone marrow, renal, cardiac and liver functions; estimated life expectancy of at least 12 weeks.

**Figure 2:**
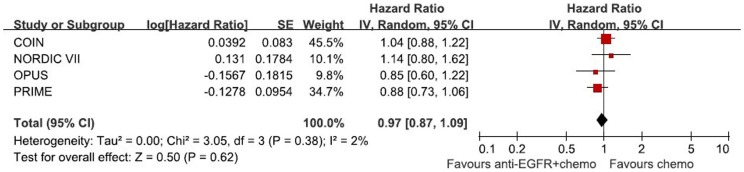
Randomized effect model on HR of OS. The pooled HR of OS is symbolized by a solid diamond at the bottom of the forest plot and the width of which represents the 95% CI.

**Figure 3:**
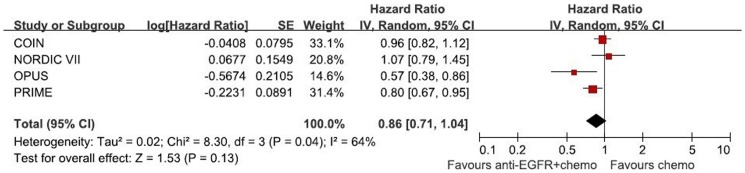
Randomized effect model on HR of PFS. The pooled HR of PFS is symbolized by a solid diamond at the bottom of the forest plot and the width of which represents the 95% CI.

**Figure 4:**
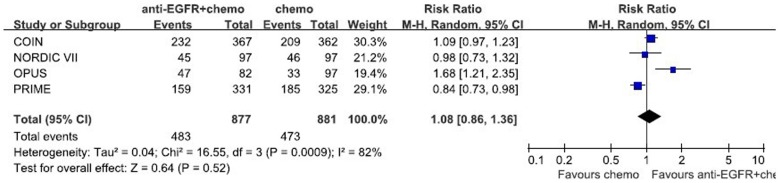
Randomized effect model on risk ratio of ORR. The pooled RR of ORR is symbolized by a solid diamond at the bottom of the forest plot and the width of which represents the 95% CI.

**Figure 5:**
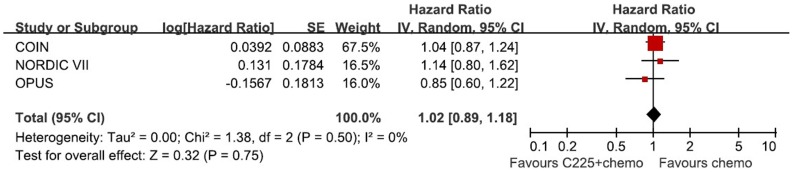
Randomized effect model on HR of OS in Cetuximab subgroup. The pooled HR of OS in cetuximab subgroup is symbolized by a solid diamond at the bottom of the forest plot and the width of which represents the 95% CI.

**Figure 6:**
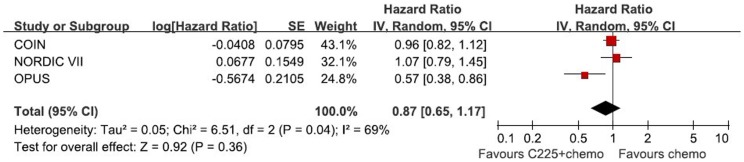
Randomized effect model on HR of PFS in Cetuximab subgroup. The pooled HR of PFS in cetuximab subgroup is symbolized by a solid diamond at the bottom of the forest plot and the width of which represents the 95% CI.

#### Types of intervention

This study evaluated oxaliplatin-based chemotherapy with or without anti-EGFR MAbs (including cetuximab and panitumumab) in the first-line treatment of mCRC. The treatment arm received anti-EGFR MAbs (cetuximab or panitumumab) combining oxaliplatin-based chemotherapy, without other targeted drugs (like bevacizumab). The control arm received oxaliplatin-based chemotherapy without any targeted drugs.

**Table 2:** Toxic effects recorded from randomized controlled trials (Grade 3–4 Adverse Events).

Studies	Intervention	Neutro-penia	Skin toxicity	Diarrhea	Thrombocytopenia	Sensory neuropathy	Fatigue
COIN [13]	FOLFOX/XELOX	13%	<1%	14%	3%	18%	18%
	FOLFOX/XELOX+Cetuximab	12%	20%	24%	3%	14%	26%
Nordic VII [14]	FLOX	47%	1%	10%	3%	22%	10%
	FLOX+Cetuximab	46%	22%	17%	4%	16%	16%
OPUS [11], [12]	FOLFOX4	34%	0.6%	7%	2%	7%	3%
	FOLFOX4+ Cetuximab	30%	11%	8%	4%	4%	4%
PRIME [15], [16]	FOLFOX4	41%	2%	9%	–	16%	3%
	FOLFOX4+Panitumumab	42%	36%	18%	–	16%	9%

#### Types of outcome measure

The primary outcome measurement was OS (death from any cause). The secondary outcomes include PFS, ORR and toxicity. The follow-up rate should be above 95%. The hazard ratio (HR), risk ratio (RR) and 95% confidence intervals (CI) of OS, PFS and response rate were directly extracted from the original studies.

**Figure 7:**
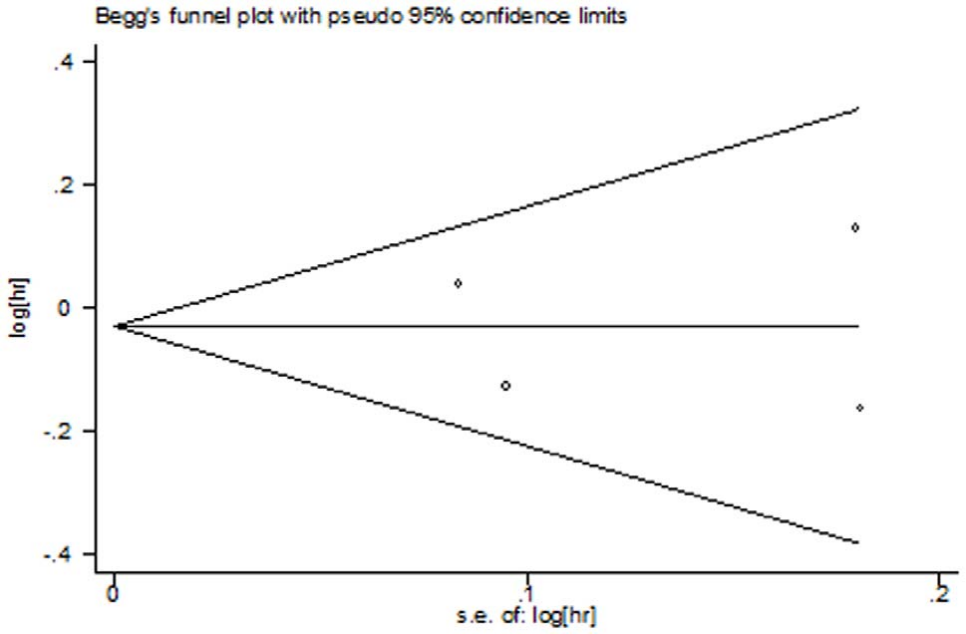
Funnel plot for publication bias test OS. The two oblique lines indicate the pseudo 95% confidence limits.

**Figure 8:**
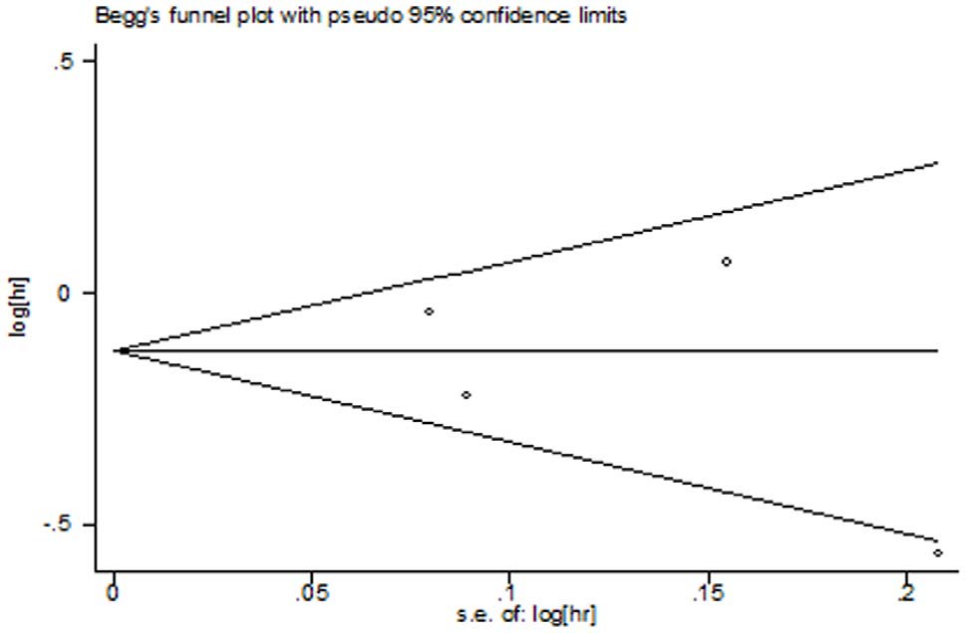
Funnel plot for publication bias test of PFS. The two oblique lines indicate the pseudo 95% confidence limits.

**Figure 9:**
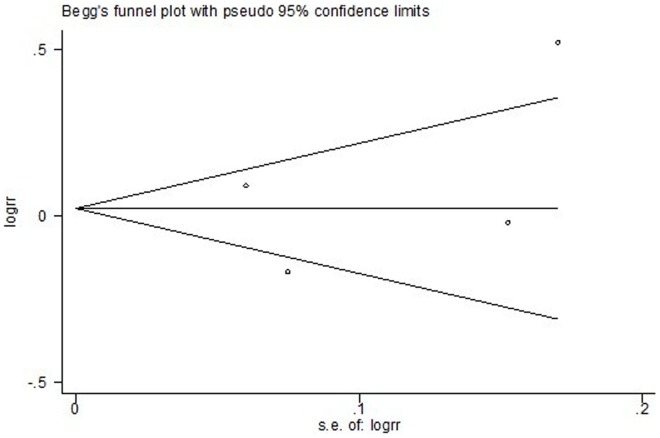
Funnel plot for publication bias test of ORR. The two oblique lines indicate the pseudo 95% confidence limits.

#### Search strategy for the identification of studies

Relevant RCTs were identified by searching electronic databases and oncology meeting websites; including Medline, EMBASE, Cochrane library, ASCO and ESMO. The latest search was done on June 28, 2012. The following subject headings and keywords were used: colorectal neoplasms, colorectal cancers, colorectal carcinomas, colorectal tumors, cetuximab, c225, MAb C225, Erbitux, panitumumab, ABX-EGF MAb, Vectibix, oxaliplatin, L-OHP, Eloxatine. For pubmed we used the search strategy as follow: ((“Colorectal Neoplasms/drug therapy”[MESH] OR “Colorectal Neoplasms/therapy”[MESH] )) AND (CETUXIMAB[Title/Abstract] OR PANITUMUMAB[Title/Abstract]) AND (OXALIPLATIN[Title/Abstract]). The language of publication was restricted to English.

#### Data extraction and synthesis

The abstracts identified from the above-mentioned sources were assessed by two independent reviewers (ZHOU Si-wei, HUANG Yuan-yuan). Both reviewers independently selected trials for inclusion according to prior agreement regarding the study population and intervention. Disagreements were resolved by consensus or by a third reviewer (XIE De-rong). Missing data from the primary study reports were requested from the investigators. If the same trial appeared on different publications, the final data of the trial were chosen. Methodological quality of the trials was assessed using a validated scale (range, 0 to 5) applied to items that influence the intervention efficacy. It was reported by Jadad et al [9] that the scale consisted of items pertinent to randomization, masking, dropouts, and withdrawals. The following information was extracted from each published trial: year of publication, first author, number of patients, performance status, chemotherapy regimen, overall response rate (ORR), OS, PFS, toxicity, follow-up period etc. For response assessment, we used trials that included patients with measurable or assessable diseases, and that were analyzed mainly with RECIST criteria. Toxicity profiles were reported according to the Common Terminology Criteria for Adverse Events (version 3.0 or 2.0).

All meta-analyses were performed using Review Manager 5.0 (RevMan 5.0; The Nordic Cochrane Centre, The Cochrane Collaboration, Copenhagen, Denmark) and Stata statistical software (release 11.0; Stata Corporation, College Station, Texas, USA). Outcomes were compared using HR and RR. Respective 95% confidence intervals (CI) were calculated for each estimate and presented in forest plots. The effect of the treatment for each single study was expressed as a ratio of the anti-EGFR chemotherapy arm over the chemotherapy alone arm.

The heterogeneity of the study results was assessed by the chi-square and I-square test, determining the use of either fixed-effects or random-effects model. Heterogeneity was defined as either a P-value<0.1 or I-square>50%. When considerable heterogeneity was detected, a possible explanation for it was pursued. When a reasonable cause was found, a separate analysis was performed. Publication bias was evaluated with the Begg’s test [10].

## Results

### Trial Flow

The flow chart of our study is demonstrated in Figure 1. Both reviewers finally agreed to include 4 trials [11]–[16] involving 1270 mCRC patients with KRAS wild type gene in the meta-analysis.

### Characteristics of the Selected Trials

These prospective RCTs are summarized in Table 1. All selected trials for inclusion strictly according to prior selection criteria, were prospective, randomized, and the clinical characteristics were matched for performance status, age, stage and gender. All studies reviewed were considered high quality, for each trial achieved a score of 3 (each point for randomization, withdrawal and appropriate method of randomization) in the assessment scale of Jadad’s study design [9]. Patients eligible for these studies had histologically or cytologically proven mCRC, with the same baseline data and without evidence of selection bias. All of the 4 trials are well organized, rigorous and prospective randomized controlled trials. The OS, PFS, ORR and toxicity data of KRAS wild type patients were extracted from 4 trials.

The OPUS study [11], [12], the only one phase II RCT in this meta-analysis, set the ORR as the primary endpoint. Unlike other 3 studies, the analysis of KRAS mutation status in this trial is retrospective. Patients were randomly assigned to the oxaliplatin-based chemotherapy, the same chemotherapy adding anti-EGFR MAbs and the intermittent chemotherapy in the MRC COIN trial [13] and the NORDIC VII trial [14]. The intermittent chemotherapy group was excluded considering the settings of same regular administration in control groups. The PRIME trial [15], [16] is the only trial regarding panitumumab, which evaluated the efficacy and safety of panitumumab plus FOLFOX4 versus FOLFOX4 alone as initial treatment for mCRC.

### Meta-analysis Results

#### OS, PFS and ORR

1270 patients from 4 randomized trials, 646 in the chemotherapy group and 624 in the chemotherapy adding anti-EGFR MAbs group, were included in the meta-analysis. Though the result of the test for heterogeneity of the therapeutic effect of 4 trials was not significant (chi-square = 2.17, P = 0.54, I^2^ = 0%), the random-effects model was used to analyze the pooled data to minimize random errors. The main result of our `meta-analysis is shown in Figure 2. Overall, no OS benefit was found from combined therapy compared to chemotherapy alone in the mCRC (HR = 1.00, 95%CI [0.88, 1.13], p = 0.95). Significant PFS benefit was not found in this study either (HR = 0.86, 95%CI [0.71, 1.04], p = 0.13). The result of PFS is presented in Figure 3. Figure 4 illustrates the results of ORR (Risk Ratio = 1.08, 95%CI [0.86, 1.36]). No significantly increasing response rate was found in the pooled analysis.

#### Subgroup analysis

Figure 5 and figure 6 show the subgroup analysis in cetuximab combination. The results reveal no significant efficacy of cetuximab combined with oxaliplatin in OS (HR = 1.02, 95%CI [0.89, 1.18], P = 0.75) and PFS (HR = 0.87, 95%CI [0.65, 1.17], P = 0.36).

#### Toxicities and safety

Toxic effects of 4 trials are summarized in Table 2 (only Grade 3–4 toxic effects were presented). Some of grade 3/4 adverse events (AEs) like skin toxicity and diarrhea were increasing by the addition of cetuximab and panitumumab to chemotherapy.

### Publication Bias

The Begg’s test and funnel plots were performed to assess the publication bias. Publication bias was defined as P-value<0.05 in Begg’s test. No evidence for publication bias was shown according to the shape of funnel plots (Fig.7, Fig 8 and Fig 9) or the Begg’s test in OS (z = 0.34, p = 0.734), PFS (z = 1.02, p = 0.308), and ORR (z = 0.34, p = 0.734).

## Discussion

The main finding of the present analysis is the combination of oxaliplatin and EGFR MAbs did not prolong OS or PFS in patients with wild type KRAS mCRC, compared with oxaliplatin-based chemotherapy alone. The addition of cetuximab or panitumumab has no statistically significant survival advantage over the single chemotherapy (HR for OS = 1.00, 95%CI [0.88, 1.13], p = 0.95; HR for PFS = 0.86, 95%CI [0.71, 1.04], p = 0.13). Panitumumab is a fully human anti-EGFR MAb, whereas cetuximab is a chimeric Mab. They are similar in mechanism of action and resistance. However, in view of different nature between two MAbs, subgroup analysis of cetuximab was conducted to confirm the efficacy. No subgroup analysis of panitumumab was performed regarding only one RCT including panitumumab. The same conclusion is found in cetuximab subgroup–no significant benefit in OS (HR = 1.02, 95%CI [0.89, 1.18], P = 0.75) and PFS (HR = 0.87, 95%CI [0.65, 1.17], P = 0.36).

The OS was set as the primary end point for several reasons. First, OS is a chief goal in the setting of incurable diseases such as mCRC. Second, OS is an objective endpoint and could be measured precisely without the influence by assessment. However, OS may be affected by crossover therapy and sequential therapy. The US Food and Drug Administration consider OS a universally accepted direct measure of treatment benefit and the end point of regular approval of drugs. PFS is not statistically validated as a surrogate endpoint for survival in all settings [17].

In our study, the reason why anti-EGFR antibodies combined with oxaliplatin-based chemotherapy did not show clinical benefit in wild type KRAS m-CRC remains unknown, while one plausible explanation is the nature and interaction of drugs used in combination. Src-family Kinases serve as a kind of non-receptor tyrosine kinases, having mutual interaction with EGFR required for proliferation, migration, survival and EGFR endocytosis. Some pre-clinical studies demonstrated that Src Kinase is activated after oxaliplatin administration through a ROS-dependent mechanism [18]. A high level of Src activates the downstream of the signal pathway of EGFR without combining ligand. In vitro study, cetuximab-resistant CRC cells showed a remarkable decrease in the level of EGFR and an enhanced role of Src kinase in collaboration with EGFR for supporting cell growth and survival [19], therefore oxaliplatin might decrease the activity of EGFR MAb.

Besides the oxaliplatin, different fluoropyrimidine regimens may also affect the efficacy of EGFR-targeted therapy differently. In the MRC COIN study [13], the predictive factor analysis shows the additional cetuximab improved the efficacy significantly in fluorouracil-based therapy while capecitabine-based subgroup has a negative result. The FOLFOX4 regimen plus panitumumab could improve PFS in the PRIME study [15], [16]. In NORDIC VII trial [14], the combination of cetuximab and FLOX regimen doesn’t prolong the OS and PFS. The FOLFOX and XELOX regimen are standard therapies in the first-line treatment of mCRC and the FLOX regimen is employed as a standard first-line regimen in the Nordic countries [14]. The efficacy of each regimen doesn’t differ significantly. It is not clear why anti-EGFR MAbs have different effects when combined with different fluoropyrimidine regimens. A possible explanation is that the addition of cetuximab resulted in reduced dose intensity (in MRC COIN study, for fluorouracil-based therapy: median 78% in the control group [Interquartile range (IQR) 70–87] vs 73% [IQR 66–82] in the cetuximab group, p = 0·031; for capecitabine-based therapy: 85% [IQR 74–92] vs 79% [IQR 67–88], p = 0·0021 [13]). It might be hypothesized that the efficacy of capecitabine-based therapy is limited by the reduction of dose intensity which was caused by more serious adverse events.

As the wide use of anti-EGFR MAbs in mCRC patients, the comprehensive and complex interaction among cytotoxic drugs, biotherapy, and patients’ gene has been observed and much importance has been attached to the appropriate selection of combined therapy. Different from other similar meta-analysis [20], [21], we directly compared the oxaliplatin-based chemotherapy with anti-EGFR MAbs to oxaliplatin-based chemotherapy alone in mCRC patients, excluding the influence of irinotecan-based regimen. Since the combination of anti-EGFR antibodies with bevacizumab might also have an impact on survival, we eliminate the PACCE and CAIRO2 study [22], [23] to see whether the addition of single anti-EGFR MAb to oxaliplatin could produce the OS benefit. Prospective or retrospective KRAS status tests were required for inclusion in this study, in order to confine to the populations who benefit from anti-EGFR MAbs (cetuximab or panitumumab) most. However, even in the KRAS wild type population, which was excluded the possible impact of patients’ gene upon anti-EGFR MAbs, no survival advantage was shown. The finding of our study demonstrates that the combination of oxaliplatin and anti-EGFR drugs didn’t prolong OS, which is at odds with irinotecan-based chemotherapy.

As to PFS, the result is more controversial because 2 of 4 trials (OPUS [11], [12] and PRIME [15], [16]) are significantly positive in PFS while the total outcome is negative. The combination of oxaliplatin and panitumumab in PRIME study benefit in PFS significantly, however, the subgroup analysis of cetuximab doesn’t show the efficacy. It’s hard to conclude that there is actually a difference between panitumumab and cetuximab in terms of PFS because of the only one RCT regrinding panitumumab. The difference, if there were any, could be attributed to several possible reasons as follows. First of all, PFS may be influenced by many factors which differ in different clinical trials, such as the definition of PFS and the intervals between evaluations. The definition of PFS and the follow-up in each enrolled trial is different. The PFS were defined as the period ranging from random assignment to first recorded progression or death in the RCTs except the OPUS study. In OPUS study, the definition of PFS is not stated clearly. In NORDIC VII, OPUS and PRIME studies, the response evaluations were conducted every 8 weeks according to the RECIST criteria. The radiologic assessment of response was carried out every 12 weeks in the MRC COIN trial. These two settings would influence the results of PFS. Secondly, the relative positive result cannot be interpreted into the benefit in the total outcome. The improvement in OPUS and PRIME studies are significant but not enough to change the total outcome. Although there seems to be a difference between panitumumab and cetuximab, the result of the test for heterogeneity of PFS was not significant (p = 0.13) and therefore the synthesis of the data is appropriate.

The pooled analysis doesn’t show the improvement of overall response rate when adding cetuximab or panitumumab in the total outcome, while the ORR in PRIME study appear significantly higher in the panitumumab arm, and this should be confirmed in more RCTs as well. The comparison of R0 resection rate was not performed since there’s no report of R0 resection rate in KRAS wild type patients in OPUS study, but the response rate can infer the limitation of anti-EGFR MAbs in the neoadjuvant therapy when combined with oxaliplatin-based chemotherapy, whereas the efficacy of panitumumab needs more evidence to be verified.

No available data were found that fatal AEs were related to cetuximab or panitumumab, though significantly increased cutaneous toxicity were observed in the combined therapies arms, attributing to heavily expressed EGFR in the skin, which may correlate to the efficacy of anti-EGFR therapy. Other increased but manageable adverse events, like diarrhea, were also reported in all trials. Considering the data of adverse events refer to intention-to-treat (ITT) population (defined as randomly assigned patients who received at least one dose of study treatment), instead of KRAS wild type patients, we did not perform a statistical analysis. In ITT population, the main AEs of anti-EGFR agents are skin toxicity and the additional biotherapy were well tolerated.

It seems that the combination of oxaliplatin and cetuximab or panitumumab didn’t show the efficacy in first-line treatment of mCRC. The result suggests that combined therapies are not just the simple addition. Each drug might have interaction with another in combination somehow by affecting the efficacy and/or toxicity. For combined therapies, drugs selection is as important as biomarker selection. Much more preclinical and clinical trials are required for combined therapies, especially the rigorous and prospective assessment. Since only one trial regarding panitumumab was included, the interpretation of efficacy of panitumumab should be more careful and more RCTs are needed to verify the conclusion.

Although this meta-analysis was based on high-quality RCTs and was properly conducted, there are some typical limitations of our study. Our findings and interpretations were limited by the quality and quantity of data available. One major limitation is the number of trials is quite small and that possibly could not unveil the real situation, but the number of patients sample is amounted to 1270. Another, all of the data were extracted from abstracted data (AD) instead of individual patient data (IPD), which would be less powerful to confirm our findings. However, a correlation analysis shows AD meta-analysis is strongly correlated with IPD meta-analysis [24], indicating AD as a kind of acceptable and practical method of meta-analysis alternative for IPD. The third, the wild type KRAS population is a subgroup of ITT population, suggesting possible selection bias. In addition, the possible existence of some unpublished studies should be aware of, which could lead to potential publication bias. However, no indication of such bias was found by using statistical methods designed to detect it. In general, regarding these limitations mentioned above, we should interpret the results with adequate caution.

In conclusion, this meta-analysis shows that the addition of cetuximab or panitumumab to oxaliplatin-based chemotherapy in first-line treatment of mCRC in patients with wild type KRAS appears no improved efficacy in survival benefit. Much more prospective clinical trials are warranted to evaluate the combination of drugs.
